# Periodontitis associated with risk of obstructive sleep apnea in Peruvian adult patients: A cross-sectional study

**DOI:** 10.4317/medoral.26561

**Published:** 2024-05-25

**Authors:** Wendy Lizbet Florian-Tirado, Angel Seven Asmat-Abanto, Delia Margarita Ulloa-Cueva, Oscar Martín Del Castillo-Huertas

**Affiliations:** 1 Orcid: 0009-0000-1231-294X. Student of Stomatology Study Program. Antenor Orrego Private University, Trujillo, Peru; 2 Orcid: 0000-0001-5726-6692. Doctor in Stomatology. Specialist in Periodontics. Professor of Human Medicine Study Program. Antenor Orrego Private University, Trujillo, Peru; 3 Orcid: 0000-0001-5726-6692. Professor of Stomatology Study Program. Antenor Orrego Private University, Trujillo, Peru; 4 Orcid: 0000-0002-6794-1652. Medical specialist in Otorhinolaryngology, associate member of the Peruvian Society of Otorhinolaryngology. Assistant physician at the Víctor Lazarte Echegaray Hospital of Trujillo, Peru; 5 Orcid: 0000-0003-0913-6361. Doctor in Stomatology. Specialist in Orthodontics and Maxillary Orthopedics. Director of the Stomatology Study Program. Antenor Orrego Private University. Trujillo, Peru

## Abstract

**Background:**

Patients at high risk for obstructive sleep apnea (OSA) are characterized by intermittent transient hypoxias and upper respiratory tract collapse, with reactive oxygen production and oxidative imbalance which causes an inflammatory cascade. This can generate negative effects on the periodontium, causing severe tooth attachment loss. This study aimed to determine the association between periodontitis and the risk of OSA in adults who attend outpatient otolaryngology consultations.

**Material and Methods:**

A cross-sectional observational study was carried out with 118 patients seen in the otolaryngology service of the Hospital Victor Lazarte Echegaray-ESSALUD in Trujillo (Peru), between September and October 2023. The presence and severity of periodontitis were determined using the Page and Eke criteria, and the presence and severity of OSA risk were determined using the STOP-BANG questionnaire. The results were analyzed using Chi-square, Spearman correlation and logistic regression tests, considering a significance level of *p*<0.05.

**Results:**

An association was found between periodontitis and risk of OSA (*p*=0.000), obtaining a positive relationship with rs=0.527. In addition, there was an association between periodontitis and DM2 (*p*=0.028) and bronchial asthma (0.017). No association was found between periodontitis and sex (0.503) or age (0.741).

**Conclusions:**

There is an association between periodontitis and the risk of OSA in Peruvian adult patients who attended outpatient otolaryngology consultations. This association was also found according to age, sex, smoking, DM2 and asthma. In addition, an association was found between periodontitis and DM2 and asthma, but not with sex and age.

** Key words:**Periodontitis, periodontal diseases, sleep apnea, hypoxia, upper respiratory tract, otolaryngology.

## Introduction

Periodontitis is a multifactorial pathology that causes chronic inflammation of periodontal tissues (1-4) and affects approximately 30-50% of the adult population worldwide, with different stages and degrees (4), and is a critical public health problem (3-6). It is caused by a dysbiosis between pathogenic bacteria and the host immune response and causes progressive destruction of the periodontium, which could result in the loss of teeth (3,4,7,8).

Numerous studies indicate that periodontitis is epidemiologically associated with several systemic disorders (9), such as diabetes mellitus (5,9), smoking (6), cardiovascular disease and obstructive sleep apnea (7), among others.

Obstructive sleep apnea (OSA) is a respiratory disorder that occurs mainly at night (10), with an overall prevalence of 3% to 7% in adults (7,11) and is characterized by the collapse of the pharyngeal respiratory tract (11,12).

OSA is caused by total or partial airflow obstruction (10), resulting in cyclic oxygen saturation, reflex sympathetic hyperactivity, poor sleep quality (12), constant snoring, awakenings with dyspnea sensation, altered muscle tone (10), blood pressure and heart rate, as well as sleep fragmentation (13). Daytime symptoms include drowsiness, headaches, asthenia, neurological disorders and impaired personal relationships (10). Consequently, patients with OSA have a poor quality of life (13).

The intermittent and transient hypoxia of OSA favors oxidative imbalance and an increase in cytokines, which triggers an inflammatory cascade (11,14,15). In addition, the presence of certain factors such as obesity, arterial hypertension, male sex, type 2 diabetes mellitus (DM2), age and metabolic syndrome will increase the risk of suffering this pathology (11,12).

Polysomnography is the main test used to diagnose OSA (10,13,16); however, taking into consideration its high cost and the prolonged duration of the procedure, questionnaires have been developed to identify patients at high risk of OSA (16,17). Among these questionnaires, the STOP-BANG has become an easy-to-use tool (11) and is composed of four clinical questions (STOP: snoring, exhaustion, observed apnea and arterial hypertension) and four demographic options (BANG: BMI, age, neck circumference and gender) (16,17).

Periodontitis and OSA, pathologies involved in systemic inflammatory reactions, have several risk factors in common; likewise, the frequency of suffering from periodontitis in subjects diagnosed with OSA is 4 times higher, indicating that both diseases could be associated with each other (7,14).

Considering the scarce research on the subject and that scientific support is required for dentists, otolaryngologists and health authorities to justify the implementation of strategies aimed at improving the oral health of patients at risk of OSA, the purpose of this study was to determine the association between periodontitis and the risk of OSA in adults who attend outpatient otolaryngology consultations.

## Material and Methods

The present study has a cross-sectional design and was carried out at the Otolaryngology Service of the Víctor Lazarte Echegaray Hospital Essalud (Trujillo, Peru), between September and October 2023. The sample consisted of 118 subjects and was calculated using the formula for independence tests, using data generated through a pilot study conducted with 40 people with the following parameters: α=0.05, β=0. 10, r=3 (Periodontitis categories), c=3 (OSA risk categories), δα,β =17.41885 (Non-centrality parameter of the Chi-square distribution, with 4 degrees of freedom). In addition, the sampling formula for Spearman's correlation coefficient was used where: Zα/2 =1.96, Zβ = 1.282 and ρ = 0.264 (Spearman's correlation estimated in the pilot sample). The selection method was non-probabilistic by convenience.

Patients between 18 and 70 years were included. Pregnant women, HIV-positive patients, partially edentulous patients with less than 6 teeth and those who did not agree to participate in the study were excluded.

The present study was approved by the Faculty of Human Medicine and the Bioethics Committee of the Antenor Orrego Private University, as well as of the Training Department of the La Libertad Assistance Network - ESSALUD, observing compliance with the principles established in the Declaration of Helsinki and the Peruvian General Health Law No. 26842.

All patients received information about the research. If they accepted to participate, they were given the informed consent form to read and sign. OSA risk was determined by the physician specialist of the service, using the STOP-BANG survey. Subsequently, the lead author assessed the presence of periodontitis according to Page and Eke's (18) criteria: severe periodontitis (two or more interproximal sites with clinical attachment loss ≥ 6 mm and probing depth ≥ 5 mm), moderate periodontitis (two or more interproximal sites with clinical attachment loss ≥ 4 mm and/or probing depth ≥ 5 mm) and no/mild periodontitis if the results were lower. In addition, basic demographic information and associated factors, such as DM2, bronchial asthma and smoking, were recorded. The history of DM2 and asthma was collected from the medical records, according to the diagnosis of the corresponding physician specialist. Concerning about the smoking habits, it was recorded according to the patient's manifestation. Previous training, the reliability for the measurement of periodontitis was determined with 10 patients, by intra- and inter-rater calibration of the principal investigator with a dental surgeon and specialist in Periodontics working at the Hospital Luis Albrecht - EsSalud (Trujillo, Peru). A Kappa coefficient equal to 1 was obtained in both calibrations.

The data collected were processed using the statistical program IBM SPSS Statistics 26.0. The association between periodontitis and risk of OSA was determined using the chi-square test of independence of criteria, Spearman's correlation was also reported. Likewise, the association between periodontitis and other factors studied was evaluated using ordinal logistic regression. A significance level of *p*<0.05 was considered.

## Results

The present study evaluated a sample of 118 patients, of whom 66.95% were male and 33.05 % were female, whose ages ranged from 18 to 70 years (X̅: 45.18; σ= 14.570).

Table 1 shows that there is an association between periodontitis and the risk of OSA (*p*=0.000). Likewise, it can be observed that 57.1% of patients at high risk of OSA presented moderate periodontitis. Regarding the prevalence of periodontitis, the no/mild group had the highest percentage (46.6%). The low risk of OSA was predominant with respect to the other risk grades (47.5%). Furthermore, due to the ordinality of the variables, the Spearman correlation was additionally analyzed, obtaining a positive relationship with rs=0.527 (*p*=0.000).

As seen in Table 2, there was an association between periodontitis and risk of OSA in both males (*p*=0.000) and females (*p*=0.021).

Likewise, it can be observed that in the male sex 50% of patients with a high risk of OSA presented severe periodontitis; while in the female sex 72.7% of patients with a high risk of OSA presented moderate periodontitis. Regarding age, the association between periodontitis and risk of OSA was also evident in patients between the ages of 18 and 40 (*p*=0.000) and in patients over 40 (*p*=0.019). Likewise, it was observed that in the 18 to 40 age group, 100% of patients at high risk of OSA presented moderate to severe periodontitis.

Regarding smoking, there was an association between periodontitis and risk of OSA, both in non-smokers (*p*=0.000) and smokers (*p*=0.016). Likewise, it can be observed that 83.3% of smokers with a high risk of OSA presented moderate periodontitis.

There was an association between periodontitis and the risk of OSA in patients without a diagnosis of DM2 (*p*=0.000), as shown in Table 3. In patients with DM2, the statistical test could not be performed due to the low number of patients enrolled. On the other hand, there was an association between periodontitis and the risk of OSA both in patients with asthma (*p*=0.014) and in patients without asthma (*p*=0.00). Likewise, in asthmatic patients, 100% of subjects with a high risk of OSA presented moderate to severe periodontitis.

Table 4 analyzed the factors under study with a multivariate model. Regarding periodontitis associated with OSA risk, patients with low (*p*=0.000) or intermediate risk (0.001) tended to present lower degrees of periodontitis than patients at high risk of OSA. Furthermore, no association was found between periodontitis and sex (*p*=0.503) or age (*p*=0.741), but an association was observed between periodontitis and the diagnosis of DM2 (*p*=0.028) and asthma (*p*=0.017), as well as with not smoking (*p*=0.009).

## Discussion

Some authors suggest that there is a possible association between periodontitis and the risk of OSA, which systemic inflammatory reactions and common risk factors could support (7,14,15,19,20). Therefore, interdisciplinary work is crucial to address these health problems comprehensively.

It was found that the prevalence of moderate to severe periodontitis increased in patients who were at high risk for OSA. Similar results were found by Mukherjee *et al*. (14). This may be due to the pathophysiological interaction between both diseases, accompanied by mouth breathing during sleep and the state of intermittent hypoxia during apnea, acting as risk factors for the development of periodontitis. Arango *et al*. (21) did not find this association either; however, patients with severe degrees of OSA had a higher tendency to present periodontitis. This author indicates that further research is required.

In this study, an association was found between periodontitis and the risk of OSA in both sexes. Similar results were reported by Keller *et al*. (22). In addition, Latorre *et al*. (15) found that females with periodontitis had a moderate risk of OSA. Similarly, a high prevalence of OSA risk was found in women with moderate to severe periodontitis. However, Mukherjee *et al*. (14) and Itzhaik *et al*. (23) found a relationship between periodontitis and the risk of OSA only with the male sex. Regarding this point, the oropharynx in males is usually larger than in females, and for this reason, sex could be expected to be an intervening variable. In view of this controversy, research with higher sample sizes is suggested, due to those that were presented, to study sex as a covariate.

It was found an association between periodontitis and risk of OSA in both age groups (18 to 40 and 41 to 70). The findings of Latorre *et al*. (15) and Ytzhaik *et al*. (23) indicate that the severity of OSA risk is associated with increasing age. According to Ytzhaik *et al*. (23), this could be due to some physiological changes, such as anteroposterior lengthening of the soft palate, an increase in parapharyngeal fat deposits, and modifications of the tissues around the pharynx. Regarding periodontitis and age, the findings of Stazić *et al*. (19) and Loke *et al*. (24) indicate that periodontitis is more severe at an older age; however, the findings of Kim *et al*. (25) did not find a relationship between age and the main variables of this study.

An association was found between periodontitis and the risk of OSA in both smokers and nonsmokers. However, Ytzhaik *et al*. (23) found that smoking, in comparison with other risk factors, is not such a determinant for developing a higher risk of OSA. This same author states that smoking, by causing irritation and localized edema in the upper respiratory tract, may contribute to the development of OSA. Likewise, Stazić *et al*. (19) found that patients who suffer more from advanced periodontitis were smokers.

In the present study, DM2 was considered a covariate and only 15 patients with this diagnosis could be evaluated. In these circumstances, there was evidence of an association between periodontitis and the risk of OSA in non-diabetic patients. The findings of Keller *et al*. (22) and Ytzhaik *et al*. (23) reveal that there is a relationship between DM2 and the risk of OSA; however, the last research team, also found that DM2 was not related to the development of periodontitis or the risk of OSA. From this perspective, reinforcing what is known, Shaktawat *et al*. (20) and Loke *et al*. (24) found an association between periodontitis and DM2.

In this study, an association was found between periodontitis and risk of OSA in patients with and without asthma. Bronchial asthma and OSA are respiratory disorders that can coexist and cause sleep disturbances, and according to some studies, the first one is associated with periodontitis. In this regard, Al-Lawati *et al*. (26) found a high prevalence of OSA in patients with bronchial asthma, which is much higher in uncontrolled cases. Moeintaghavi *et al*. (27) also found a relationship between periodontitis and asthma; this could be a consequence of the low salivary flow and the characteristics of the oral microbiome in asthmatic patients due to drugs used in these patients.

Multivariate analysis allows different variables and their effect on each other to be studied simultaneously. For this reason, to enhance the present study, Wald ordinal logistic regression was used, and it was found that there was no difference with respect to the severity of periodontitis according to the sex and age group of the patients. Additionally, it was found that there was a higher predisposition to develop severe periodontitis as the patients presented a higher risk of OSA, reinforcing what was found in the bivariate analysis. Likewise, periodontitis is associated with DM2 and bronchial asthma. However, as an atypical finding, it was found that non-smoking patients presented a higher frequency of periodontitis, which could be because smoking was studied as a covariate. There were only 21 patients with this characteristic. Consequently, increasing the sample size is suggested in subsequent studies to provide more information about this finding.

One limitation of the present study is how the DM2 and smoking covariates were measured. Both were measured dichotomously; in the case of DM2, whether or not the medical diagnosis was present and, in the case of smoking, whether or not the patient smoked. Different results could be obtained if it had been classified as a prevalent or incident case of DM2 because the diagnosis of long-standing DM2 can affect periodontal health to a greater extent. It would also be essential to classify smokers according to the number of cigarettes consumed per day since greater periodontal involvement is expected in patients with greater consumption. We suggest increasing the number of patients evaluated in future studies to enrich the results and better understand the role of these covariates.

As this is a cross-sectional study, this research provides preliminary evidence because it does not have temporal control between variables. For this reason, it is recommended to perform longitudinal studies, to add information or challenge these findings, thus determining possible causal associations.

Based on the results obtained, it is suggested that the otolaryngologist may refer the patient with a diagnosis or risk of OSA to the dentistry area for evaluation, treatment and periodontal control. Likewise, the dentist can use the STOP-BANG questionnaire to detect the presence of OSA risk and refer the patient to the otolaryngologist for treatment. It is necessary to promote interdisciplinary work to address these two pathologies, considering the clinical guidelines and the information provided to improve the quality of life of these patients.

## Figures and Tables

**Table 1:** Association between periodontitis and risk of OSA in Peruvian adult patients of the outpatient otolaryngology consultation.

Risk of apnea	Periodontitis							X^2^	*p*
	No/mild		Moderate		Severe		Total		
	N^o^	%	N^o^	%	N^o^	%			
Low	37	66.1	19	33.9	0	0.0	56	-	-
Medium	17	41.5	18	43.9	6	14.6	41	-	-
High	1	4.8	12	57.1	8	38.1	21	-	-
Total	55	46.6	49	41.5	14	11.9	118	33.829	0.000

Nº: number of patients, %: percentage of patients, X^2^: Chi-square test of independence of criteria, p: significance level.

**Table 2:** Association between periodontitis and risk of OSA in Peruvian adult patients of the outpatient otolaryngology consultation, according to sociodemographic factors.

Socio-cultural factors		Risk of apnea	Periodontitis							X^2^	*p*
			No/mild		Moderate		Severe		Total		
			N^o^		%	N^o^	%	N^o^	%		
Sex	Male	Low	30	69.8	13	30.2	0	0.0	43	23.029	0.000
		Medium	13	50.0	8	30.8	5	19.2	26		
		High	1	10.0	4	40.0	5	50.0	10		
		Total	44	55.7	25	31.6	10	12.7	79		
	Female	Low	7	53.8	6	46.2	0	0.0	13	11.561	0.021
		Medium	4	26.7	10	66.7	1	6.7	15		
		High	0	0.0	8	72.7	3	27.3	11		
		Total	11	28.2	24	61.5	4	10.3	39		
Age	18-40 years old	Low	24	72.7	9	27.3	0	0.0	33	25.804	0.000
		Medium	2	50.0	2	50.0	0	0.0	4		
		High	0	0.0	4	50.0	4	50.0	8		
		Total	26	57.8	15	33.3	4	8.9	45		
	41-70 years old	Low	13	56.5	10	43.5	0	0.0	23	11.845	0.019
		Medium	15	40.5	16	43.2	6	16.2	37		
		High	1	7.7	8	61.5	4	30.8	13		
		Total	29	39.7	34	46.6	10	13.7	73		
Smoke	No	Low	27	62.8	16	37.2	0	0.0	43	27.139	0.000
		Medium	15	38.5	18	46.2	6	15.4	39		
		High	1	6.7	7	46.7	7	46.7	15		
		Total	43	44.3	41	42.3	13	13.4	97		
	Yes	Low	10	76.9	3	23.1	0	0.0	13	12.216	0.016
		Medium	2	100.0	0	0.0	0	0.0	2		
		High	0	0.0	5	83.3	1	16.7	6		
		Total	12	57.1	8	38.1	1	4.8	21		

Nº: number of patients, %: percentage of patients, X2: Chi-square test of independence of criteria, p: significance level.

**Table 3:** Association between periodontitis and risk of OSA in Peruvian adult patients in outpatient otolaryngology consultation, according to associated diseases.

Diseases		Risk of apnea Group	Periodontitis							X^2^	p
			No/mild		Moderate		Severe		Total		
			N^o^		%	N^o^	%	N^o^	%		
DM 2	Yes	Low	1	25.0	3	75.0	0	0.0	4	33.745	0.000
		Medium	3	42.9	2	28.6	2	28.6	7		
		High	0	0.0	3	75.0	1	25.0	4		
		Total	4	26.7	8	53.3	3	20.0	15		
	No	Low	36	69.2	16	30.8	0	0.0	52		
		Medium	14	41.2	16	47.1	4	11.8	34		
		High	1	5.9	9	52.9	7	41.2	17		
		Total	51	49.5	41	39.8	11	10.7	103		
Bronchial asthma	Si	Low	4	50.0	4	50.0	0	0.0	8	12.444	0.014
		Medium	0	0.0	8	88.9	1	11.1	9		
		High	0	0.0	2	50.0	2	50.0	4		
		Total	4	19.0	14	66.7	3	14.3	21		
	No	Low	33	68.8	15	31.3	0	0.0	48	26.957	0.000
		Medium	17	53.1	10	31.3	5	15.6	32		
		High	1	5.9	10	58.8	6	35.3	17		
		Total	51	52.6	35	36.1	11	11.3	97		

Nº: number of patients, %: percentage of patients, X^2:^ Chi-square test of independence of criteria, p: significance level.

**Table 4:** Ordinal regression analysis of periodontitis and risk of OSA and other cofactors in Peruvian adult patients of the otolaryngology outpatient consultation.

Factors			Coefficient	Standard error	Wald	*p*
Threshold	Periodontitis	No/mild	-1.093	0.799	1.871	0.171
		Moderate	2.009	0.793	6.417	0.011
Labelling	Sex: male		-0.324	0.484	0.448	0.503
	Age: 18-40 years old		-0.153	0.464	0.109	0.741
	Smoke: No		1.786	0.680	6.906	0.009
	Apnea	Low	-3.227	0.648	24.795	0.000
		Medium	-2.571	0.642	16.014	0.000
	DM2: Yes		1.432	0.650	4.851	0.028
	Asthma: Yes		1.345	0.563	5.699	0.017

Wald: Wald test for logistic regression, p: significance level.
